# Photovoltaic Properties in Interpenetrating Heterojunction Organic Solar Cells Utilizing MoO_3_ and ZnO Charge Transport Buffer Layers

**DOI:** 10.3390/ma3114915

**Published:** 2010-11-08

**Authors:** Tetsuro Hori, Hiroki Moritou, Naoki Fukuoka, Junki Sakamoto, Akihiko Fujii, Masanori Ozaki

**Affiliations:** Division of Electrical, Electronic and Information Engineering, Graduate School of Engineering, Osaka University, 2-1 Yamada-oka, Suita, Osaka 565-0871, Japan

**Keywords:** organic solar cells, interpenetrating heterojunction, buffer layers, molybdenum trioxide, zinc oxide

## Abstract

Organic thin-film solar cells with a conducting polymer (CP)/fullerene (C_60_) interpenetrating heterojunction structure, fabricated by spin-coating a CP onto a C_60_ deposit thin film, have been investigated and demonstrated to have high efficiency. The photovoltaic properties of solar cells with a structure of indium-tin-oxide/C_60_/poly(3-hexylthiophene) (PAT6)/Au have been improved by the insertion of molybdenum trioxide (VI) (MoO_3_) and zinc oxide charge transport buffer layers. The enhanced photovoltaic properties have been discussed, taking into consideration the ground-state charge transfer between PAT6 and MoO_3_ by measurement of the differential absorption spectra and the suppressed contact resistance at the interface between the organic and buffer layers.

## 1. Introduction

Since the discovery of photoluminescence quenching [1], photoinduced charge transfer [2], and photoconduction enhancement [3,4] in conducting polymers (CPs)-fullerene (C_60_) systems, donor-acceptor type organic solar cells based on π-conjugated polymers have been investigated as next-generation solar cells. Two typical device structures, a layered structure [5] and a CP-C_60_ composite structure [6], are mainly studied. However, a semi-layered structure with an interpenetrating interface has also been suggested as a candidate structure for obtaining high efficiency [7,8].

In the semi-layered structure, photoinduced charge transfer occurs efficiently because of the large interface area between donor and acceptor layers, and the generated electrons and holes can be transported efficiently to the electrodes. Previously, we developed a simple, yet effective method of fabricating such an interpenetrating interface, and reported an improvement in the photovoltaic conversion efficiency [9,10,11,12,13,14]. For the interpenetrating heterojunction type organic solar cell, we have reported buffer layer effects with the insertion of an appropriate metal oxide layer between the organic layer and the metal electrode, such as zinc oxide (ZnO) or molybdenum trioxide (VI) (MoO_3_) [10,13,14]. We demonstrated the optimum film thicknesses of ZnO and MoO_3_ layers, which act as electron and hole transport buffer layers, respectively. However, the studies about interpenetrating heterojunction type organic solar cells using both buffer layers and the mechanism of the photo conversion enhancement still remains to be carried out.

In this paper, we report on the improvement in the photovoltaic properties of the interpenetrating heterojunction solar cells upon the insertion of both MoO_3_ and ZnO buffer layers and discuss about the enhanced photovoltaic properties.

## 2. Experimental

**Figure 1:**
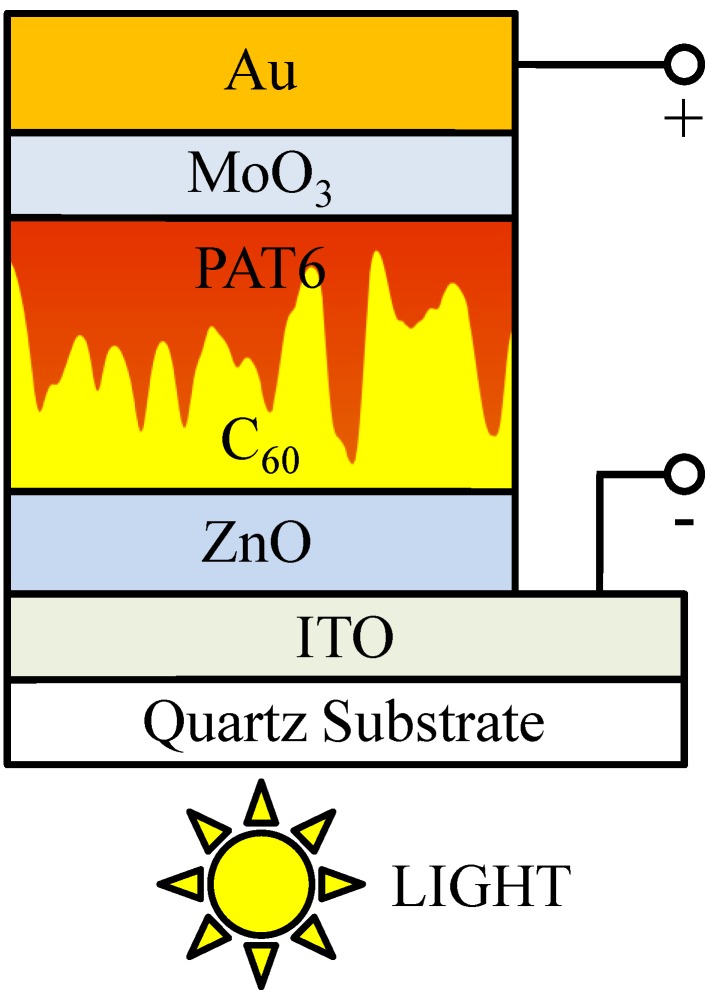
Schematic structure of interpenetrating heterojunction organic thin-film solar cell with metal oxide buffer layers.

The solar cell structure of indium-tin-oxide (ITO)/ZnO/C_60_/poly(3-hexylthiophene) (PAT6)/MoO_3_/Au, as shown in Figure 1, was fabricated in the following manner. A ZnO layer with a thickness of 50 nm [10] was sputtered onto ITO-coated quartz substrates. A C_60_ film with a thickness of approximately 130 nm was deposited by thermal evaporation onto the ZnO layer under 10^−^^4^ Pa at a substrate temperature of 150 °C [12]. The chloroform solution of PAT6, the concentration of which was 16.6 mg/mL, was spin-coated onto the C_60_ layer at 500 rpm for 5 sec and 1500 rpm for 30 sec. A MoO_3_ layer and an Au electrode were fabricated by thermal evaporation through a shadow mask onto the PAT6 layer under a pressure of 10^−^^4^ Pa. The evaporation rates of MoO_3_ and Au were approximately 0.3 Å/s and 1.0 Å/s and the thicknesses of the MoO_3_ layer and Au electrode were 6 nm [13] and 70 nm, respectively. The active area of the solar cell was 1 × 2 mm^2^. In the cells, the ITO and Au electrodes collected electrons and holes, respectively.

The current-voltage characteristics were measured with a high-voltage-source measurement unit (Keithley 237) under AM1.5 (100 mW/cm^2^) solar-illuminated conditions. From the current-voltage characteristics under AM1.5, the fill factor (FF) and energy conversion efficiency (η_e_) were estimated using FF = *I*_max_*V*_max_*/I*_sc_*V*_oc_ and η_e_ = *I*_sc_*V*_oc_FF*/P*_in_, where *I*_max_ and *V*_max_ are the current and voltage, respectively, for the maximum output power, *I*_sc_ is the short-circuit current density, *V*_oc_ is the open-circuit voltage, and *P*_in_ is the intensity of the incident light. The external quantum efficiency (EQE) spectra were measured under the short-circuit condition using an electrometer (Keithley 617S) and xenon lamp light passing through a monochromator as a light source. The current-voltage characteristics and EQE spectra were then measured in vacuum at room temperature. The EQE was estimated using EQE(%) = *I*_sc_ × 124,000 / (λ(nm) × *P*_in_), where λ is the wavelength of incident light. The UV-VIS-NIR absorbance spectra were measured using a spectrophotometer (Shimadzu UV-3150).

## 3. Results and Discussion

**Figure 2:**
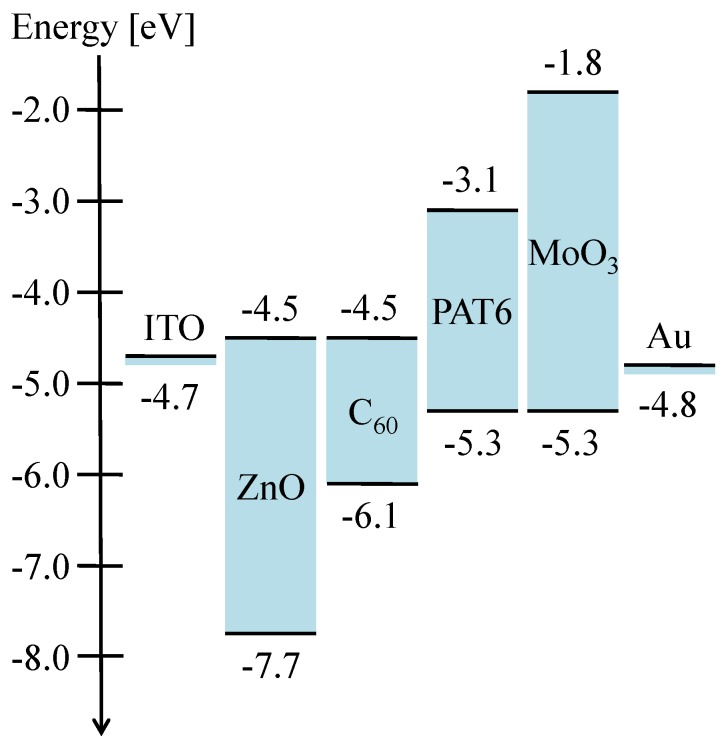
Schematic energy levels of ITO, ZnO, C_60_, PAT6, MoO_3_, and Au.

The energy diagram of the solar cell with the ITO/ZnO/C_60_/PAT6/MoO_3_/Au structure is shown in Figure 2. According to the energy level of MoO_3_, a negligible energy barrier for holes and a high energy barrier for electrons exist at the interface with PAT6. On the other hands, ZnO exhibits a negligible energy barrier for electrons and a high energy barrier for holes at the interface with C_60_. In addition, since the buffer layers and adjacent electrodes form ohmic contacts, the internal electrical field intensity of the organic layers increases upon inserting buffer layers, therefore, it is expected that *V*_oc_ increases.

**Figure 3:**
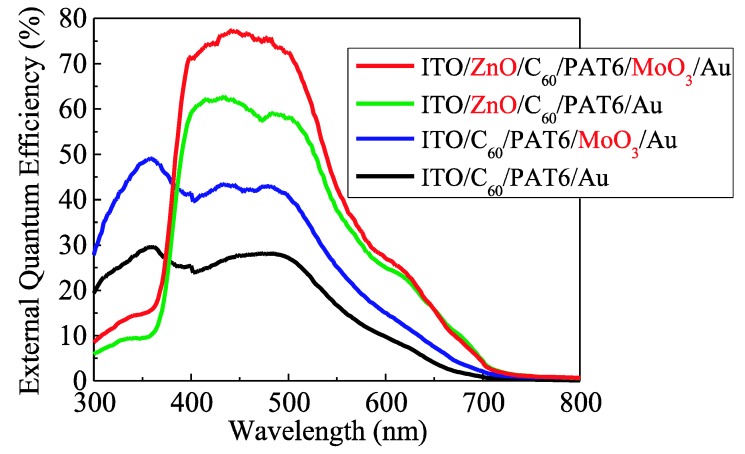
EQE spectra of the solar cells with no buffer layer, the MoO_3_ buffer layer (6 nm), the ZnO buffer layer (50 nm), and the MoO_3 _and ZnO buffer layers (6 nm and 50 nm, respectively).

Figure 3 shows the EQE spectra of the solar cells with no buffer layer, the MoO_3_ hole buffer layer, the ZnO electron buffer layer, and both buffer layers. The EQE of the solar cell with the MoO_3_ hole buffer layer was about 40%, and that with the ZnO electron buffer layer was about 60% in the visible region. The EQE of the solar cell with the ZnO buffer layer decreased in the UV region, because the ZnO layer absorbs UV light and plays the role of a light filter. The EQE of the solar cell with both buffer layers increased further and exceeded 75%.

**Figure 4:**
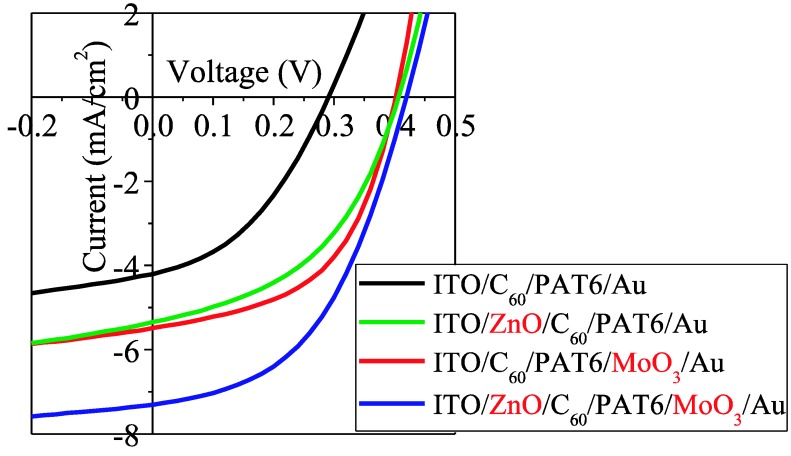
Current-voltage characteristics of the solar cells with no buffer layer, the MoO_3_ buffer layer (6 nm), the ZnO buffer layer (50 nm), and the MoO_3 _and ZnO buffer layers (6 nm and 50 nm, respectively) under AM1.5 (100 mW/cm^2^) solar-illuminated conditions.

The current-voltage characteristics of the same solar cells under AM1.5 (100 mW/cm^2^) solar-illuminated conditions are shown in Figure 4, and each performance parameter is summarized in Table 1. It is considered that each buffer layer enables similar improvements since comparable *V*_oc_ and *I*_sc_ values were obtained in the solar cells inserted with one of the buffer layers. The MoO_3_ buffer layer was more effective than the ZnO buffer layer in terms of the FF. *I*_sc_ was further enhanced, and *V*_oc_ also increased slightly in the solar cell with both buffer layers. As a result, η_e_ was enhanced about three times from 0.49% to 1.46% by inserting both MoO_3_ and ZnO charge transfer buffer layers.

**Figure 5:**
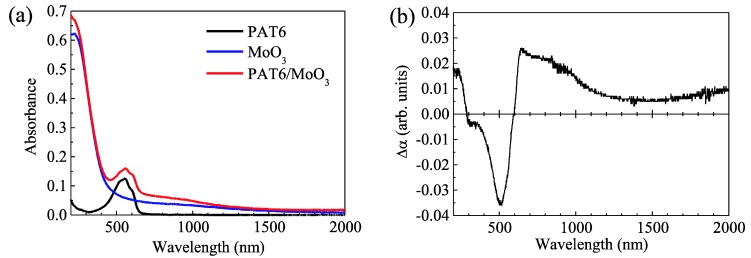
(**a**) UV-VIS-NIR absorbance spectra of the PAT6 film, MoO_3_ film, and PAT6/MoO_3_ bi-layer, film and (**b**) differential absorption spectrum.

In order to discuss the interface between the PAT6 and MoO_3_ layers, the UV-VIS-NIR absorption spectra of the PAT6 film, MoO_3_ film, and PAT6/MoO_3_ bi-layer film were measured, and the differential absorption spectrum, which is obtained by subtracting the absorption spectra of the PAT6 and MoO_3_ films from that of PAT6/MoO_3_ bi-layer film, was investigated, as shown in Figure 5. In these measurements, thinner PAT6 films were prepared compared with the PAT6 layers in the solar cells, the thickness of which was estimated to be 20 nm, in order to clarify weak signals from the interface of PAT6/MoO_3_. The absorbance range of 400−600 nm, which is based on the π-π* transition in PAT6, was decreased, and the absorbance in the near-infrared region appeared upon the formation of the interface of the PAT6 and MoO_3_ layers. A similar phenomenon was previously found to be the result of an insulator-metal transition of a CP such as PAT6 upon electrochemical doping [15]. It is noted that the ground-state charge transfer at the interface between the PAT6 and MoO_3_ layers occurred to form polarons in the region near the interface. Therefore, it is considered that the conductivity of PAT6 partially increased near the interface with the MoO_3_ layer or the contact resistance was suppressed, resulting in enhancement of the FF. 

**Table 1:** Performance parameters in current-voltage characteristics of the solar cells with no buffer layer, ZnO buffer layer (50 nm), MoO_3_ buffer layer (6 nm), and ZnOand MoO_3_ buffer layers (50 nm and 6 nm, respectively) under solar-illuminated conditions.

Structure of the solar cells	*V*_oc_ [V]	*I*_sc_ [mA/cm^2^]	Fill Factor	η_e_ [%]
ITO/C_60_/PAT6/Au	0.29	4.21	0.40	0.49
ITO/ZnO/C_60_/PAT6/Au	0.41	5.35	0.45	0.99
ITO/C_60_/PAT6/MoO_3_/Au	0.40	5.49	0.52	1.15
ITO/ZnO/C_60_/PAT6/MoO_3_/Au	0.42	7.31	0.48	1.46

Although a similar measurement of the differential absorption spectrum of ZnO/C_60_ bi-layer films was also carried out, such a marked spectral change was not observed at this stage. From the measurement of the conductivity of the ZnO film, the conductivity under dark and AM1.5 solar-illuminated conditions are found to be 10^-13^ S/cm and 10^-8^ S/cm, respectively. Since the ZnO film absorbs the UV light included in solar-illumination, and bulk electron traps in ZnO tend to be filled, the marked photoconduction of ZnO could be observed [16,17]. Therefore, it is considered that the efficient electron collection and the suppression of *V*_oc_ loss by leak current blocking in the solar cell were realized by inserting a 50-nm-thick ZnO buffer layer.

## 4. Conclusion

Interpenetrating heterojunction organic solar cells with MoO_3_ and ZnO charge transport buffer layers were demonstrated. We achieved enhancement of the photovoltaic properties of interpenetrating heterojunction type organic solar cells by inserting a MoO_3_ hole buffer layer and a ZnO electron buffer layer. As a result, the EQE of the solar cells exceeded more than 75% and the energy conversion efficiency was improved from 0.49% to 1.46% under AM1.5 solar-illuminated conditions. We discussed the photovoltaic properties in terms of the ground-state charge transfer and reduced the contact resistance at the interface between organic and buffer layers.
